# A Review on Extraction, Characterization, and Applications of Bioactive Peptides From Pressed Black Cumin Seed Cake

**DOI:** 10.3389/fnut.2021.743909

**Published:** 2021-09-01

**Authors:** Ahmed A. Zaky, Jae-Han Shim, A. M. Abd El-Aty

**Affiliations:** ^1^Department of Food Technology, National Research Centre, Cairo, Egypt; ^2^Natural Products Chemistry Laboratory, College of Agriculture and Life Sciences, Chonnam National University, Gwangju, South Korea; ^3^State Key Laboratory of Biobased Material and Green Papermaking, College of Food Science and Engineering, Shandong Academy of Science, Qilu University of Technology, Jinan, China; ^4^Department of Pharmacology, Faculty of Veterinary Medicine, Cairo University, Giza, Egypt; ^5^Department of Medical Pharmacology, Medical Faculty, Ataturk University, Erzurum, Turkey

**Keywords:** black cumin seed cake, antioxidant peptides, functional attributes, antioxidant activity, applications, safety

## Abstract

Plenty of black cumin cake was generated as a natural waste material after pressing the oil. *Nigella sativa* (black cumin) seeds and cakes are of precious nutritional value as they contain proteins, phenolics, essential amino acids, and bioactive compounds. Owing to their antioxidant properties, scientists and food manufacturers have extensively developed them. Notably, global awareness among consumers about the benefits of innovative food ingredients has been increased. Meanwhile, it has to be noted that vast amounts of cake by-products are not effectively utilized, which might cause economic loss and environmental consequences. This review aimed to highlight the antioxidant abilities, extraction, characterization, functional characteristics, and utilization of active peptides acquired from black seed oil cake. This overview would critically evaluate black seed cake proteins, plentiful in bioactive peptides that might be utilized as valuable additives in feed, food, pharmaceutical, and cosmetic industries. The addition of bioactive peptides to restrain the oxidation of fat-based products and preserve food safety is also addressed.

## Introduction

Presently, awareness toward the use of medicinal plants in the prevention and treatment of different diseases has received considerable attention because they are safe with minimal or no side effects. According to the WHO, more than 60% of the population of the world relies on traditional medicine for primary healthcare (1). Furthermore, the WHO has supported developing countries to use natural remedies available and accessible in their vicinity to design novel healthcare programs (2).

Black cumin or black seed (*Nigella sativa*) is an annual herbaceous plant of the family Ranunculaceae. It is cultivated in several parts of the world, especially the Middle East (3, 4). Black seeds are utilized as an essential spice, flavoring agent, and medicinal herb. It shows a mean composition of 38.20% fat, 20–85% proteins, 7–94% crude fibers, 31.94% carbohydrates, 4% ash, and 5% moisture (5, 6). In the last decades, black seed has been used as a medicinal plant, owing to its actual impacts on humans to cure serious health problems (3, 7). In this context, various nutraceutical attributes have been associated with black seeds, such as antioxidant, anticancer, antiinflammatory, antiallergic, antimicrobial, and antifungal properties (8–10).

Black cumin seeds used for oil extraction produced a vast amount of cake as a significant by-product that remains underutilized mainly (11). Cumin seeds cake is an excellent source of protein, fibers, carbohydrates, vitamins, phenolic, and antioxidants that may benefit human health (12, 13).

Overall, there is a lack of systematic reports on exploiting proteins isolated from black cumin press cake to gain novel bioactive compounds. So far, multiple reports have formally addressed the importance, benefits, and functional properties of black cumin seeds (Table 1). Herein, we focused on all aspects that were not reviewed elsewhere by others, namely, characterization, extraction, purification, identification, functional properties, amino acid profile, and application and safety profiles of pressed black cumin seed cake.

**Table 1:** Comparison of current aspects with other published reviews on black cumin seeds (*Nigella sativa*).

**References**	**Source**	**Covered aspects**
(14)	Cumin seed (*Nigella sativa)*	Pharmacognostic characteristics, chemical composition, and pharmacological activity of the seeds of cumin.
(15)	Black cumin oilseeds	Nutritional value, functional attributes, and nutraceutical applications of black cumin oilseeds.
(16)	Black cumin	Phytochemical profiles, health benefits, molecular pharmacology, and safety of black cumin.
(17)	*Nigella sativa seed*	Dermatological effects of *Nigella sativa* seed.
(18)	Black cumin essential oil	Biological activity, such as antifungal, antibacterial, and antioxidant potentials, as well as food application.
(11)	Black cumin	Overview of the nutritional, therapeutic, and biomedical applications and prospects of black cumin seeds in feeding humans, animals, and poultry.
(19)	*Nigella sativa*	Pharmacological impacts, such as antibacterial, antiviral, antiinflammatory, and wound-healing effects.
(20)	*Nigella sativa*	The role of *Nigella sativa* and its bioactive components in learning and memory.
Current study	Black cumin seed and its cakes	- Characterization, extraction, functional properties of antioxidant proteins and peptides from black cumin seed and its cakes. - Amino acid profile of black cumin and its cakes. - Application of bioactive compounds (such as proteins, peptides, and polyphenols) in feed, food, pharmaceutical, and cosmetic industries. - Nutritional and safety aspects.

In this review, eligible studies (in English) were recognized throughout an electronic search of the PubMed database (1994–2021) (https://www.nlm.nih.gov/) and Google. We used the primary search term “Black seed cake proteins” connected with terms “antioxidant peptides,” “antioxidant activity,” “extraction,” “characterization,” “purification,” “identification,” “amino acid profile,” “functional properties,” “application,” and “safety” to figure out the appropriate literature. We screened the titles, keywords, and abstracts of publications gained from the database. If considered appropriate, full-text articles were collected for accurate evaluation.

## Black Cumin Seeds Cake Protein

Protein-energy undernutrition is a common form of protein deficiency, demonstrating a significant problem in developing countries. Presently, the vast crowds are confronting a lack of adequate healthy food, causing scarcity of macronutrients. Proteins, lipids, vitamins, minerals, and carbohydrates are required in sufficient quantities to maintain good health; therefore, their lack may lead to unanticipated consequences (21). Consequently, a serving of protein-rich food is required for growth and body action. Nonetheless, protein intake is the interventional approach to reduce the burden of malnutrition (22). Thus, discovering low-cost, high-quality protein is a crucial mission for food scientists (23). In this respect, oilseed cake or deoiled part could effectively be employed as an essential source of protein to enhance the food products (24). The oilseed cakes protein has typical chemical properties that may enhance the nutritional and functional characteristics of the food products. Black cumin seeds have widely been utilized in traditional culinary and medicinal purposes. Furthermore, several studies have suggested that black seed cake possesses potential benefits, namely, high protein quality with different biological activities (25, 26). Moreover, defatted black seed cake protein serves as a good source of minerals, namely, Mg, P, Na K, and Ca (27). Thence, oilseed cake may be utilized as food supplements, as it contains approximately 12% protein with no side effects on growth or blood characterization (26).

## Characterization of Black Cumin Seeds and Their Cakes

Black cumin seed is the second important cash crop utilized in conventional items. Notably, its utilization in various food products has increased in recent years because of its health benefits. It is mainly grown on small plots of land in several parts of the world. As a result, its global production has yet to be adequately recorded (28). The black cumin has medicinal properties attributed to bioactive ingredients, such as thymol, thymoquinone (TQ), nigellicine, nigelline, and carvacrol (29). In addition, black seed may have chemopreventive effects against different types of cancer.

Concerning minerals, black seeds contain a relatively higher amount of phosphorus, calcium, and iron than magnesium, zinc, manganese, and copper (30, 31). Furthermore, Cheikh-Rouhou et al. (32) compared the chemical compositions of two types of black cumin (Tunisian and Iranian). They found that moisture, fat, protein, ash, and carbs concentrations were 8.65, 28.48, 26.7, 4.86, and 40.0%, in Tunisian and 4.08, 40.35, 22.60, 4.41, and 32.70% in Iranian type, respectively.

Cumin seed cake is a prime food by-product having a high biological value protein and minerals. According to the findings of Abdel-Magid et al. (25), the seed cake is a valuable source of rough protein and nitrogen-free extract, with 37.40 and 35.81%, respectively. Moreover, Attia et al. (33) found 27.90–34.10% protein contents in black cumin cake. Similarly, Aydin et al. (34) stated that the *Nigella sativa* cake is versatile with 30% protein and essential amino acids. Formerly, Bewley et al. (35) confirmed that Arg, Asp, and Glu exist in the seeds of black cumin as major amino acids, while Cys and Met are secondary amino acids.

## Extraction of Proteins From Cumin Seed Cake

Vegetable-derived proteins and their component bioactive peptides have been shown to possess health-promoting properties, owing to their nutritional value. Therefore, a series of processes are used to separate protein derived from different sources (Table 2). According to Horax et al. (36), protein solubility in organic solvents, followed by elimination and purification using the appropriate approaches, is the most common method. In a physical method, cells exposed to acute stress showed degradation of cell walls; in turn, proteins are easily liberated. Nevertheless, physical techniques give lower extraction yields than chemical and enzymatic strategies. Therefore, depending on the solubility behavior of protein fractions, various types of separation processes were used for diverse protein sources, such as grains, oilseeds, and legumes (37).

**Table 2:** Advantages and disadvantages of standard methods used to extract proteins from black cumin seed cake.

**Methods**	**Characterizations**	**Advantages**	**Disadvantages**	**References**
Physical extraction	As a rule, cell wall degradation is conducted by acute stress to the cell structure.	- Improves the liberation of protein. - In the industry, this technique is more economical and easy to adapt and use.	Lower extraction yields than other techniques.	(36–38)
Alkaline extraction	Alkaline extraction followed by isoelectric precipitation is the most common and conventional technique for obtaining high-yield protein extracts, as it can efficiently break hydrogen and amide bonds to solubilize proteins.	- Alkaline conditions are especially effective in extracting proteins from plant sources (i.e., black cumin seed cake). - Simple and inexpensive to provide protein isolates.	- Protein denaturation. - Extraction of nonprotein components that are co-precipitated with proteins. - Accelerating Maillard reaction, resulting in dark-colored products.	(38–41)
Micellization	This strategy is used for the recovery of protein isolates that involve precipitation by sodium chloride in cold water.	In this process, the dissolution step separates a small insoluble portion that is not recovered by isoelectric precipitation.	- Starch is the major contaminant of protein isolates.	(42–45)
Enzyme-assisted extraction	Enzyme-assisted extraction is a common approach to produce value-added protein isolates. Enzymes are used in different ways to promote protein extraction in black cumin cake, such as cell wall degradation, starch-bond protein release, and protein solubility improvement.	- Enzymatic hydrolysis does not affect the nutritional value of proteins. - Substantial enhancement in protein recovery can be accomplished when enzymes are used.	The use of commercial enzymes is very costly.	(38, 46, 47)

Alkaline extraction is a well-known technique for obtaining high-yield protein extracts from plant sources. Protein isolates and concentrates for the food sector can be prepared *via* an alkaline-based method followed by precipitation at an isoelectric point. Proteins are essentially soluble in an alkaline solution and subsequently precipitated in an acidic medium (38). Protein extracts from deoiled cakes were commonly carried out with NaOH (mild alkaline conditions) at a pH range of 8–10. The solid sediments, especially carbs, are then removed from the solubilized proteins using centrifuging. Subsequently, the obtained proteins can be separated from residue remained after solubilization using isoelectric precipitation (dilute acid solution, HCl) followed by centrifuging (39). Finally, the sediment was washed to eliminate the residual soluble elements. The conventional alkaline method is versatile because it is inexpensive and straightforward to provide protein isolates. The plainness of the method and cost-effective chemicals make it a practical option. Unfortunately, though, ultrafiltration membrane and salting-out through aqueous mitigation are a little costly (40, 41).

Micellization is also one of the protein extraction procedures acknowledged as a salt removal method. It differs from other techniques based on the salting-in and salting-out process. In this approach, the required salt concentrations denature the protein at a specific ionic force, diluting the solution. Centrifugation followed by drying was used for protein precipitation. NaCl dissolved in ice water was used for precipitation. The dissolution phase separates a tiny insoluble fraction, which could not be obtained through isoelectric precipitation (42, 43). As a result, the micellization process had lower albumin content than other fractions, such as glutelin, globulin, and prolamin. Still, starch is the most common impurity in protein concentrates (44). The lower amount of albumin in protein is because of high starch solubility (45).

Enzyme-assisted extraction is a common technique to produce value-added protein isolates. Hanmoungjai et al. (46) reported that digestion of proteases increases the functional attributes of concentrates, thus achieving the right texture in products, such as infant formulas, beverages, and nutraceuticals. This approach uses various enzymes to ensure that the obtained protein does not contain unsafe levels of contaminants. For example, according to Wang et al. (47), xylanase and phytase enzymes were used for protein digestion and to break down carbs. The protein concentrates acquired after enzymatic digestion possessed a 74.5–92.0 g/100 g protein content and 36.1–74.6 g/100 g yield.

The enzymatic alteration improves the functionality and application range of protein concentrates. The peptides formed *via* partial protein digestion have low molecular mass and smaller secondary structures than the native ones. Although an inadequate degree of hydrolysis was perceived throughout the separation of soy protein during particular proteases, namely, pepsin, trypsin, papain, and chymotrypsin, a greater degree of hydrolysis can be achieved by a mixture of endoenzymes and exoenzymes. Furthermore, unextractable substances (protein and carb) can be extracted through a commercial enzyme preparation containing proteinase and carbohydrase (48). From the experience of the author, the enzymatic methodology is the best approach for extracting bioactive peptides. The black cumin seed cake acts as possible storage for protein retrieval; though, there is a lack of information regarding protein separation and fortification in the edibles.

## Purification and Identification of Bioactive Peptides

All approaches adopted for the purification and identification of all bioactive peptides are almost the same. Purification of bioactive peptides should be performed to obtain an economically viable product. The purification of bioactive peptides can be carried out by ultrafiltration, reversed-phase high-performance liquid chromatography, size exclusion chromatography, and ion-exchange chromatography. Furthermore, analytical methods based on mass spectrometry (MS); electrospray ionization/MS; matrix-assisted laser desorption/ionization time-of-flight/MS; liquid chromatography-MS/MS; and hydrophilic interaction liquid chromatography (HILIC) are routinely used for protein identification.

## Functional Properties

The functional attributes are among the physicochemical features that would affect the behavior of raw materials and final products throughout processing, storage, and usage (35). Some factors, such as molecular mass, amino acid formation, net charge, structure, hydrophobicity, and molecular hardness, in addition to the external conditions (temperature, pH, and salt concentration) or reaction with other food ingredients (49), might impact the functional behavior of proteins in foods. Furthermore, the textural and functional attributes of protein concentrates are modified depending on the techniques used for isolation (50).

The nitrogen solubility is principally based on the physical and chemical properties of protein particles in addition to other functional characteristics, such as foaming, gelling, and emulsification attributes (51, 52). Trigui et al. (53) reported a significant difference (*P* < 0.05) in the foaming capacity and the foaming stability of Tunisian and Turkish black cumin protein samples using the different concentrations (0.01–0.05 g/ml). Results revealed that the Tunisian purified black cumin protein had better foaming properties than the Turkish one. This finding might be attributed to their superior interfacial behavior at the air–water interface.

The water and oil absorption traits of protein concentrates could impact the physical force, flexibility, plasticity, and final product stream. The absorption features also benefit other protein properties, namely, wettability, swelling, and water holding capacity (38, 54). The black cumin cake protein has excellent functional behavior with efficient water and oil retention and good emulsifying properties, thus increasing the product quality. The resultant cake is a more economical nutrient-dense source that minimizes the cost of composite blends and final edibles besides enhancing the nutritional values (55). The absorption characteristics of plant proteins play a vital part in flavor preservation and mouthfeel (56, 57).

Emulsification is a vital process, which is often used to evaluate protein-rich foods. Overall, emulsifying characteristics of black cumin cake protein are influenced by molecular volume and elasticity, enhanced by decreasing the formation of intra- and intermolecular disulfide bonds and solubility (47). In this respect, Lorenz and Sikorski (52) found that the emulsifying activity index of legume proteins might be affected by surface hydrophobicity because the hydrophobic amino acids expose remain throughout the emulsion production. Increasing protein solubility would promote emulsifying attributes in salt-prepared protein solutions (53). In this context, Trigui et al. (53) declared that increasing the temperature from 25 to 95°C would boost the emulsifying capacity index and the emulsion stability index for isolated proteins from Tunisian and Turkish black cumin seeds.

Gelation is another important functional property of globular protein needed to alter food texture depending on the extraction process (58). The presence of ionic species and other factors, such as heating rate and temperature, and pH, play a vital role in affecting the gelation network of black cumin proteins. These operating factors affect gel structure (59). In addition, the decrease in proteolysis capacity is necessary to maintain the gelation attributes of separated proteins, which would otherwise lead to rough texture gel (60, 61). Based upon the aforementioned details and the experience of authors, the outstanding functional properties of black cumin seeds proteins make them beneficial for food applications.

## Antioxidant Activity

Peptides derived from black seed (its cakes) proteins show promising health-enhancing bioactivity. Herein, we focused on the antioxidant capacity of bioactive peptides recovered from food products and wastes materials.

Lipid oxidation represents the most critical chemical changes throughout the production, storage, and final preparation of fatty foods (62). The final products of lipids oxidation deteriorate the color, odor, flavor, and texture of food products, diminishing the nutritional benefits and shelf life (63).

Antioxidants play an essential role in decreasing oxidative reactions in both food models and the human body. In fatty food-based systems, antioxidants can inhibit lipid peroxidation and other unpleasant components formation (64). Artificial antioxidants, namely, butylated hydroxyanisole (BHA), butylated hydroxytoluene, propyl gallate, and tert-butyl hydroquinone, the most widely used ones, have the power to decrease the growing threat of the oxidation process in fatty foods (65). Nonetheless, the application of artificial additives in food processing has been reducing due to uncertain carcinogenic impacts and rejecting the products by the consumer (66). In this regard, replacing synthetic antioxidants with natural ones may offer health benefits and functionality (67). There is a trend of applying antioxidant peptides/proteins to restrain oxidation issues in lipids or fatty foods.

## Antioxidant Peptides, Proteins, and Amino Acids

Among a wide variety of antioxidants used in food products, antioxidants peptides have attracted considerable attention. TQ is an antioxidant phytochemical compound found in black cumin seed and its cakes. Screening the antioxidant activity of black cumin essential oil using thin-layer chromatography identified radical scavenging characteristics for TQ (68, 69). TQ played a central role in protecting organs from oxidative damage induced by reactive oxygen species. Moreover, Vasilchenko et al. (70) isolated thionins (well-known as antioxidant and antimicrobial peptides) from defatted black seeds and their cakes using acidic extraction accompanied by precipitation using cooled acetone. Additionally, Sabow (71) reported that peptide antioxidants could be formed during the enzymatic hydrolysis of various proteins. Some antioxidant peptides, such as β-lactoglobulin, ovalbumin, and soybean protein, have been identified in hydrolysates of diverse proteins to scavenge free radicals in goat meat.

Several studies have reported the reverse association between the incidence of diseases and dietary intake of antioxidants. The antioxidant ability of peptides might be attributed to inhibition of lipid peroxidation, chelating metal ions, and radical scavenging characteristics. It has also been confirmed that the structure of peptides and amino acid sequence could affect their antioxidative potency (72). Amino acids, namely, Met, Lys, His, Tyr, Gly, Trp, and Pro, are principally categorized as antioxidants (73, 74), fortunately, present in black seed cake protein. In addition, some of the amino acids possess the antioxidant capacity that could be attributed to their concentrations and pH of the media. Furthermore, Rajapakse et al. (75) documented that amino acids with aromatic residues can donate protons to electron-deficient radicals. This mechanism might contribute to the radical-scavenging properties of amino acids.

On the other hand, iron-binding proteins and antioxidant enzymes are used to prevent lipid oxidation in fatty foods. Those proteins enhance the antioxidant capacity of some food products (76). The gross antioxidant capability of proteins could be increased by interrupting its tertiary structure, which eventually boosts the solvent suitability for an amino acid to remain. More free radicals and prooxidative chelate metals are scavenged. The peptide formed through the hydrolyzing process became the primary applicable technique for producing antioxidant proteins, where peptides possessing superior antioxidant activity contrasted to the whole proteins (77). From our experience, peptides and proteins have massive prospects as antioxidants for food, mainly lipids or fatty food.

## Amino Acid Profile of Black Cumin and Its Cakes

The health-improving capacity of protein is defined by the characteristic, amount, and allocation of essential amino acids in the final product (78). Unfortunately, plant-based proteins are inadequate in essential amino acids, such as Lys, Ile, Met, Val, and Try, where they eventually have a reduction in the content and/or are entirely devoid of them. Hence, to replace large quantities of meat protein in a product with proteins from plants, two or three types of raw vegetable materials with complementary amino acid compositions should be supplemented to offer the organism all exogenous amino acids *via* the complementation system (79).

The essential amino acids are vital for function and must be provided in sufficient quantity by nourishment. A comparison of the essential amino acid composition of black seed cake with selected oilseeds, beef, and chicken meat is given in Table 3. The amino acid profile of black seeds is nutritionally valuable as it contains the essential amino acids, consistent with the Food and Agriculture Organization/WHO standards (15, 39). The black cumin is abundant in essential amino acids, such as Leu, Lys, Arg, Phe, and Glu. In this context, Abd El-Rahman et al. (86) found that the amino acid level in black seed cake was higher than in the Jatropha seed cake. The primary amino acids in black seed cake are Glu, Cys, Arg, Asp, Leu, Ala, Tyr, and Phe. At the same time, another work declared that black cumin is plentiful in glutamic acid than arginine. The cumin cake protein owns plenty of aromatic amino acids (Phe and Tyr) with registered chemical scores of 122 (76, 80, 87). From our experience, essential amino acids possess excellent prospects as antioxidants for food.

**Table 3:** Essential amino acid composition of the black seed cake, oilseeds, and raw meat (g/100 g).

**Amino acid**	**Leu**	**Ile**	**Lys**	**Val**	**Met**	**Tyr**	**Phe**	**Thr**	**Trp**	**AA sum**	**References**
Black seed cake	1.11	0.64	0.63	0.82	0.23	0.54	0.64	0.63	0.12	5.36	(80)
Chia seeds	1.35	0.73	0.98	0.93	0.80	0.58	1.10	0.76	0.79	8.02	(81)
Rapeseed (meal)	2.51	1.25	2.04	1.55	0.47	0.99	1.44	1.59	0.43	12.27	(82)
Sunflower seeds	1.40	0.92	0.86	1.11	0.53	0.57	1.05	0.81	0.35	7.6	(83)
Soybean seeds	3.47	1.97	2.37	1.94	0.59	1.35	2.25	1.63	0.57	8.02	(84)
Flaxseed	1.18	0.87	0.75	1.07	0.32	0.53	0.95	0.72	0.30	6.69	(85)
Pumpkin seeds	2.49	1.29	1.36	1.71	0.73	1.19	1.81	1.04	0.61	12.23	(83)
Beef (raw, lean, 3% fat)	1.71	0.97	1.82	1.08	0.57	0.68	0.86	0.85	0.11	8.65	(85)
Chicken (raw breast, skinless)	1.86	1.10	2.16	1.16	0.58	0.81	0.91	1.01	0.28	9.87	(85)

## Application of Black Cumin and Its Cakes

### Feed Applications

Black seed cake (obtained by cold pressing) is a valuable by-product employed in numerous sectors. Since it has a large amount of protein and carbohydrates, it can be used in animal feed. The final nutritional value and protein digestibility of the product are developed by substituting 30–60% soybean protein with cumin seed cake (25). In this regard, El-Gaafarawy et al. (88) noted a meaningful increase in gross feed consumption, expected weight growth, and feed conversion rate when the meal was substituted with black seed cake. Furthermore, El-Ayek et al. (89) reported that black cumin cake is a cost-effective alternative to replace 50% of the protein in fodder formulas. Similar trends were recorded by El-Deek et al. (90), who confirmed that black cumin cake protein could be utilized up to 50% in the broiler chicks feeding with no side impact on growth, meat quality, feed eating, conversion rate, and safety.

Additionally, Zeweil et al. (26) evaluated the impact of black cumin cake on the growth parameters, dietary digestion, and blood properties of white New Zealand rabbits by partial or complete substitution with soybean cake. They used 0, 6, 12, and 24% of the black cumin cake. They found that rabbits fed on 12% black cumin cake gained more overall weight than the control group, with a 3.7% increase in feed conversion rate. A mix of black seed cake protein with corn cake has been studied throughout a bioassay in another study. The resultant diet significantly increased serum protein, albumin, and globulin levels with no adverse effects (80). Furthermore, black cumin cake could be applied as a protein and energy supplement in the feed of growing lambs instead of soybean meal and barley (91).

### Food Applications

In the food sector, antioxidant proteins and peptides could be employed as alternatives for synthetic antioxidants, as they have comparable or even better ability for restraint of lipid peroxidation (92–94). Nonetheless, few reports were provided to evaluate the influence of antioxidant peptides for their potential use as additives in real foods (Table 4).

**Table 4:** Application of bioactive compounds obtained from black seed and its by-products in the food industry.

**Source**	**Application**	**References**
Black seed oil	Minced meat (prevent lipid oxidation)	(95)
Black cumin ethanolic extracts	Chicken meatballs (reducing lipid oxidation)	(96)
Black seed cake extract	Inhibiting corn oil oxidation	(12)
Black cumin	Cheese (enhancing nutritional value)	(97)
Black cumin essential oil	Enhance the oxidative stability of butter	(98)

Meat products are considered more sensible to lipid oxidation than other foodstuffs and need extra protection by reducing or inhibiting the formation of reactive species. Antioxidant proteins in meat-based products aid in protecting cells from harm induced by oxidative stress *via* radical scavenging, metal ion binding, or the renewal of different oxidized antioxidants (94). Although the peptides have antioxidant power, they may affect the action of antioxidant enzymes leading to enhanced efficiency. In this context, Mahgoub et al. (95) stated that the shelf-life of ground beef enriched with cold-pressed black seed oil was prolonged due to the slow growth of pathogenic microorganisms. After 15-day storage under cold conditions, the impedance to the development of *Salmonella enteritidis* was greater than that of *Listeria monocytogenes* in meat supplemented with 4% black seed oil.

Similarly, black cumin ethanolic extracts were combined with chicken meatballs at 1.2% of the meat mixture in another study. The oxidative alterations were reduced through cold storage due to greater total phenolic content and more substantial radical scavenging capacity (96). Furthermore, peptides were acquired from cottonseed albumin, producing great antioxidant features in meat-based foods (99). Accordingly, Bougatef et al. (100) isolated and identified peptide antioxidants using the enzymatic digestion of round *Sardinella* proteins. The authors confirmed that the obtained peptides possessed excellent antioxidant capacity in fat-based foods.

Furthermore, Mariod et al. (12) investigated the oxidative stability of corn oil at 70°C with black seed cake extract (0.25 and 0.5%) in the darkness and compared it with BHA. In comparison to control and BHA, the inclusion of extract resulted in decreased peroxide and anisidine levels. They also stated that the inclusion of 0.25% from black seed cake as a typical antioxidant effectively inhibited corn oil oxidation than 0.5%. This concentration would be higher from the marketing side. Phenolic compounds of black seed cakes own potent antioxidants to protect the seeds from damage by invasive pest species (101). The inclusion of black cumin, in the case of seeds, oil, and cakes, is being employed in the formulation of functional dairy products, such as cheese, where the portion of cumin ingredients contributed to the enhancement of the nutritional significance of the product, development of the antioxidant efficacy, expand their shelf-life, and, most importantly, meet the consumer preference (100).

Moreover, according to Sabbah et al. (102), black biodegradable/edible protein-based films were made from deoiled black seed cake. Proteins in the examined defatted cake can serve as transglutaminase acyl donor and acceptor substrates being polymerized when produced *in vitro* using the enzyme. The obtained results establish the soundness of the approach to recognize the cumin cakes as protein-based renewable origins to provide compost, animal fodder, or table food and added-value products, such as bioplastics.

Regarding all the nutritional information and the experience of the authors, it is proposed that black seed cake protein concentrates be included in the food preparations. Hence, supplementing diet staples with black seed cake is safer for the weak slice to afford high-quality protein.

### Pharmaceutical Applications

In pharmaceutical applications, black cumin seed and its by-products have been employed as anticancer and antiinflammatory agents, and others (Table 5). Regarding anticancer activity, the cytotoxic impacts of various black seed samples as an adjunct treatment to doxorubicin on human MCF-7 breast cancer cells were assessed. The research revealed that the LC50 of black seed lipid extract is 2.720 ± 0.2 mg/ml, which means it is cytotoxic to MCF-7 cells. In contrast, when the concentration was as high as 50 mg/ml, the cytotoxicity of the aqueous extract was apparent (103). Furthermore, the administration of black cumin was found to decrease the carcinogenic influences of dimethyl-benz(a)anthracene carcinogen in mammary carcinoma, demonstrating the significance of black cumin in treating mammary carcinoma (104). Moreover, in animal studies, Shuid et al. (105) found that the aqueous extract of black seed owned antiinflammatory and analgesic properties but not antipyretic properties. Additionally, black cumin oil has an excellent radioprotective effect against ionizing immunosuppressive and oxidative impacts of radiation (107).

**Table 5:** Application of bioactive compounds obtained from black seed and its by-products in the pharmaceutical industry.

**Source**	**Application**	**References**
Black seed extracts	Anticancer	(103)
*Nigella sativa* seed	Anticancer	(104)
Black seed (aqueous extract)	Antiinflammatory	(105)
*Nigella sativa* seed	Antiinflammatory	(106)
Black seed oil	Immunosuppressive agent	(107)
Cumin seed extract	Antibacterial	(108)
Cumin seed extract	Antiviral	(17)
Black seed (ether extract)	Antifungal	(109, 110)
Cumin seed oil	Antivitiligo	(111)
*Nigella sativa* fixed oil	Wound healing agent	(112)
Black seed	Antiulcer agent	(113)
Black seed (aqueous extract and oil)	Antidiabetic	(114)

Drug-resistant microbes are a tackling threat and global healthcare problem. Reports on the influences of cumin seed extract *in vitro* against resistant microorganisms, such as resistant Staphylococcus *aureus* and Pseudomonas *aeruginosa*, showed outstanding results vs. much multidrug-resistant gram-positive and gram-negative bacteria (108). In addition to enhancing immunity, cumin seed extract possessed some restrained impact on the human immunodeficiency virus protease; however, the main reason(s) accountable for this action was not recognized (17, 115). Concerning the antifungal activity, TQ was proved to have a mild action against clinical isolates of dermatophytes (trichophyton, epidermophyton, and microsporum); the ether extract of black seed was also efficient but at a relatively higher level (109). Similar trends were recorded by Khan et al. (110), who found that the ether extract of black seed inhibits the growth of *Candida* yeasts in different tissues of infected laboratory animals. In a randomized paired-blind clinical research, patients who utilized cumin seed oil two times a day for 6 months possessed a notable reduction in the vitiligo zone scoring index without any substantial adverse impacts (111). Ahmed et al. (116) found that black cumin and its oil would improve wound healing in experimental mice. In this context, Abu-Al-Basal (112) mentioned that black cumin (ether extract) employed topically onto staphylococcal-infected skin in rats heightened healing by decreasing whole and absolute differential white blood cell counts, topical infection and inflammation, bacterial development, and tissue damage.

### Cosmetic Applications

According to Amin et al. (117), the characteristic features of emulsions prepared with the black seed cake extracts (*in vivo* and *in vitro*) have been assessed using pH meter, corneometer, methyl nicotinate pattern of microinflammation in human skin, and tape stripping of the *Stratum corneum*. The authors confirmed that emulsions with black seed cakes significantly diminished skin soreness and increased skin moisture and epidermal barrier role compared to placebo. Consequently, the authors proposed that cumin cakes could be used for antiaging, moisturizing, and mitigation.

## Safety of Black Seed and Its Cakes

Various toxicological investigations have been carried out on black seed and its cakes. No harmful effect was noticed when black seed fixed oil was administered to mice through oral gavage. In a chronic toxicity work, mice given daily oral dosage for 3 months showed no alterations in liver enzymes, especially alanine-aminotransferase, aspartate-aminotransferase, and gamma-glutamyl transferase. Furthermore, the histopathological examination of the heart, kidneys, pancreas, and liver tissues revealed no changes. In rats, the LD50 values of black seed fixed oil after single oral ingestion and intraperitoneally were 26.2–31.6 and 1.86–2.26, respectively. The lower toxicity of black seed fixed oil demonstrated by high LD50 values, stability of liver enzymes, and organ integrity assumed a broad safety margin for black seed fixed oil (118). Yagoub et al. (119) stated that adding black cumin (10 or 15%) to meals of rabbits would have no significant impact on serum protein and albumin/globulin (A/G) concentrations; nonetheless, adding 20% gave a gradual decrease. Similar trends were noticed by Hassan et al. (120) and Tollba and Hassan (121), who found no significant differences amongst control and black cumin-supplemented groups based on the A/G ratio.

On the other hand, Al-Jishi and Hozaifa (122) demonstrated that a black cumin cake-supplemented diet increased blood total protein and A/G ratios of mice. Kidneys excrete creatinine, urea, and uric acid, the by-products of protein metabolism; however, a significant rise in these elements denotes renal failure (123). In this regard, Zeweil et al. (26) documented that serum urea concentrations were 28, 20, and 30 mg/dl in rabbits feed on different amounts of black cumin. Also, Tousson et al. (124) found that rabbits given a black cumin diet had urea and creatinine levels of 65.87 and 1.52 mg/dl, respectively. Additionally, Eapen et al. (125) found that plasma urea concentrations in female and male rats varied from 13.7 to 15.2 and 12.1 to 14.3 mg/dl, respectively. Creatinine levels varied from 0.30 to 0.41 mg/dl in females while, male Sprague–Dawley rats possessed creatinine concentrations ranged from 0.36 to 0.39 mg/dl (126, 127). Overall, normal serum urea and creatinine levels demonstrated the safety of black cumin protein isolates based on diets.

## Conclusions and Future Perspectives

The present review highlighted the functional attributes, health benefits, and antioxidants of active proteins obtained from black cumin cake as a by-product after pressing the oil. The extraction, characteristics, and amino acid profile of black seed cake proteins were also addressed. The black seed cake protein can be effectively applied in planning more functional foods and probably can be used as a food additive in fatty foods to improve their shelf-life and oxidative stability. The development of novel antioxidant peptides from black seed protein for feed, food, pharmacological, and cosmetic applications was also reviewed. Furthermore, possible uses of the acquired biomaterials from black cumin cakes (such as coating/wrapping of various foods to extend shelf-life) should be evaluated. Notably, some of the bioactive peptides have already been marketed; nonetheless, there is a desire to figure out the novel origins from which new peptides could be isolated and used as bioactive components in lipid-based foods. Therefore, further studies are required to assess the antioxidant capability, bioavailability, and health impacts of present and undiscovered bioactive peptides.

## Author Contributions

AZ and AA contributed significantly to analysis and manuscript preparation. AZ supported valuable discussion. AZ, J-HS, and AA revised the whole manuscript. All authors collated papers, wrote the manuscript, and read and approved the final manuscript.

## Conflict of Interest

The authors declare that the research was conducted in the absence of any commercial or financial relationships that could be construed as a potential conflict of interest.

## Publisher's Note

All claims expressed in this article are solely those of the authors and do not necessarily represent those of their affiliated organizations, or those of the publisher, the editors and the reviewers. Any product that may be evaluated in this article, or claim that may be made by its manufacturer, is not guaranteed or endorsed by the publisher.
